# Rolling Circle Amplification Tailored for Plasmonic Biosensors: From Ensemble to Single-Molecule Detection

**DOI:** 10.1021/acsami.2c14500

**Published:** 2022-11-29

**Authors:** Katharina Schmidt, Simone Hageneder, Bernadette Lechner, Barbara Zbiral, Stefan Fossati, Yasaman Ahmadi, Maria Minunni, Jose Luis Toca-Herrera, Erik Reimhult, Ivan Barisic, Jakub Dostalek

**Affiliations:** † Biosensor Technologies, AIT-Austrian Institute of Technology GmbH, Konrad-Lorenz-Straße 24, 3430 Tulln an der Donau, Austria; ‡ CEST Competence Center for Electrochemical Surface Technologies, 3430 Tulln an der Donau, Austria; § Molecular Diagnostics, Health & Environment, AIT Austrian Institute of Technology GmbH, 1210 Vienna, Austria; ∥ Department of Nanobiotechnology, University of Natural Resources and Life Sciences Vienna (BOKU), 1190 Vienna, Austria; ⊥ Department of Chemistry “Ugo Schiff”, University of Florence, via della Lastruccia 3-13, Sesto Fiorentino, 50019 Firenze, Italy; # FZU-Institute of Physics, Czech Academy of Sciences, Na Slovance 2, 182 21 Prague, Czech Republic

**Keywords:** rolling circle amplification, surface plasmon resonance, surface plasmon-enhanced fluorescence, biosensor, single molecule, immunoassays

## Abstract

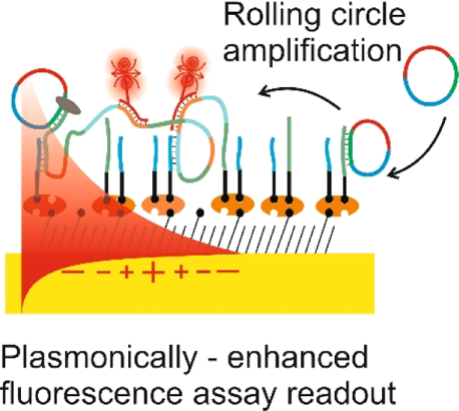

We report on the tailoring of rolling circle amplification (RCA) for affinity biosensors relying on the optical probing of their surface with confined surface plasmon field. Affinity capture of the target analyte at the metallic sensor surface (*e.g.*, by using immunoassays) is followed by the RCA step for subsequent readout based on increased refractive index (surface plasmon resonance, SPR) or RCA-incorporated high number of fluorophores (in surface plasmon-enhanced fluorescence, PEF). By combining SPR and PEF methods, this work investigates the impact of the conformation of long RCA-generated single-stranded DNA (ssDNA) chains to the plasmonic sensor response enhancement. In order to confine the RCA reaction within the evanescent surface plasmon field and hence maximize the sensor response, an interface carrying analyte-capturing molecules and additional guiding ssDNA strands (complementary to the repeating segments of RCA-generated chains) is developed. When using the circular padlock probe as a model target analyte, the PEF readout shows that the reported RCA implementation improves the limit of detection (LOD) from 13 pM to high femtomolar concentration when compared to direct labeling. The respective enhancement factor is of about 2 orders of magnitude, which agrees with the maximum number of fluorophore emitters attached to the RCA chain that is folded in the evanescent surface plasmon field by the developed biointerface. Moreover, the RCA allows facile visualizing of individual binding events by fluorescence microscopy, which enables direct counting of captured molecules. This approach offers a versatile route toward a fast digital readout format of single-molecule detection with further reduced LOD.

## Introduction

Analysis of deoxyribonucleic acid (DNA) strands has become an irreplaceable tool in biomedical diagnosis^1,2^ and other bioanalytical fields.^3^ In addition, these molecules constitute essential building blocks serving in other areas spanning from nanotechnology^4^ to synthetic biology^5^ and genetic engineering.^6^ Such progress was in particular enabled by the development of various techniques for DNA amplification, including polymerase chain reaction (PCR),^7^ rolling circle amplification (RCA),^8^ and loop-mediated amplification.^9^ For the detection of DNA molecules in liquid samples, these methods serve in conjunction with microfluidic systems^10^ and microarray technologies^11,12^ for multiplexed detection of trace amounts of DNA^13−15^ as well as for DNA sequencing.^16^

Since PCR relies on repeated thermal cycles, alternative isothermal reactions were pursued to simplify the amplification. Among these, RCA became an established amplification method for the detection of DNA sequences which is typically performed in aqueous bulk solutions.^8^ Literature has reported various modifications to RCA improving the sensitivity of assays, including the addition of restriction enzymes in circle-to-circle amplification,^17,18^ multiple hybridization of primers,^19^ and hyperbranched RCA,^20^ as well as their applications in DNA origami,^21,22^ DNAzymes,^5^ sequencing,^23^ and sensitive protein detection.^24^ In the quest for ultrasensitive detection, RCA was also used for single-molecule detection by using fluorescence imaging.^25,26^ In such assays, the capture of the target analyte is employed to initiate the synthesis of long single stranded DNA (ssDNA) chains decorated with a high number of fluorophore tags. The presence of the target analyte is then manifested as individual bright spots at locations where the fluorescently labeled RCA product is generated.^27^ This gives rise to the possibility of facile implementing of the so-called digital readout format based on direct counting of target molecules. This can be achieved by partitioning the volume of the analyzed sample using, for example, microdroplet generators,^28^ microfluidic wells,^29^ or separation of captured species with magnetic beads, into arrays of reaction chambers.^30^ Moreover, RCA can be implemented on solid surfaces by anchoring ssDNA probes for multiplexed assays.^31^ This approach also paved the way for the partitioning-free digital readout of the assay in conjunction with the fluorescence imaging readout.^32^ Research on manipulating the long RCA-generated DNA chains by introducing short DNA staples to form a compact globular conformation improved resolving the sensor signal of individually captured target molecules.^33^

An RCA step performed on solid surfaces can be utilized for sensitive detection of chemical and biological species by using various transducing principles including electrochemical, gravimetric, or optical methods (overview with typical analytical performance characteristics presented in Supporting Information, Section S1).^34−38^ Among them, using the metallic surface allows for efficient optical probing with the evanescent field of surface plasmons (SPs).^39−42^ These optical waves originate from collective oscillations of charge density and an associated tightly confined electromagnetic field. In surface plasmon resonance (SPR)-based biosensors, a ligated circular ssDNA strand (padlock probe) can be docked on the sensor surface *via* the affinity capture target analyte to initiate RCA and thus increase the measured refractive index changes.^39^ In addition, the SP optics can serve for the amplification of weak fluorescence signals. In surface plasmon-enhanced fluorescence (PEF) spectroscopy, SPs are employed for probing the surface where affinity binding of fluorophore-tagged molecules occurs.^43,44^ In conjunction with RCA, the presence of the target analyte on the sensor surface can be associated with a high number of fluorophore tags introduced by additional hybridization of repeating motifs on long RCA chains with respective complementary sequences labeled with fluorophores. In conjunction with the PEF readout, this route offers dual amplification means for highly sensitive readout of affinity binding events.

Our previous work investigated the growth of several tens of micrometer long ssDNA chains generated by RCA on a gold surface with high grafting density by using the combined SPR and PEF method.^45^ Here, we follow up on this work by studying the impact of conformation of RCA-generated ssDNA chains on the dual enhancement efficiency (optical and enzymatic) when it is applied for detecting a low concentration of the target analyte. When decreasing the analyte concentration, the transition from densely packed to sparsely attached individual DNA strands occurs on the surface. This effect is detrimental for the optical response strength of plasmonic biosensors that allow for the monitoring only within the evanescent field of SPs reaching ∼100 nm from the sensor surface. The utilization of ionic strength change and affinity interaction-based guiding along the biointerface are explored to compress the RCA-generated ssDNA chains toward the surface. Based on the control of the distance of the fluorophore tags from the sensor surface, fluorescence enhancement in the PEF readout is maximized, and consequently the limit of detection (LOD), when detecting an ensemble of target molecules, is improved. In addition, the possibility of detection of individual binding events with fluorescence microscopy is tested in order to further push this analytical performance characteristic, and possible implementation to sandwich immunoassays is presented.

## Experimental Section

### Materials

The oligo(ethylene glycol) (OEG)–thiols [OEG–OH, HS–(CH_2_)_11_–EG_6_–OH, prod. no. TH 001-m11.n6; OEG–biotin, HS–(CH_2_)_11_–EG_6_–biotin, prod. no. TH 004-m11.n6; OEG–COOH, HS–(CH_2_)_11_–EG_6_–OCH_2_–COOH, prod. no. TH 003-m11.n6] were obtained from ProChimia Surfaces (Poland). Phosphate-buffered saline (PBS, pH = 7.4, cat. no. E504), nuclease-free water (NFW, cat. no. E476), Tween 20 (cat. no. 437082Q), and 99.9% pure ethanol (cat. no. 1.11727) were from VWR (Austria). As a working buffer (PBST), there was used PBS with 0.05% (v/v) Tween 20. Calcium chloride (CaCl_2_, cat. no. C1016), sucrose (cat. no. S7903), Trizma-hydrochloride solution (Tris-HCl, pH 8.0, 1 M, cat. no. T2694), dimethylsulfoxide (DMSO, 99.9% pure, cat. no. 41640-M), dibenzocyclooctin-*N*-hydroxysuccinimidylester (DBCO-NHS, cat. no. 761524), and ethanolamine (1 M in water, adjusted to pH 8.5 with NaOH and HCl, cat. no. E9508) were purchased from Sigma-Aldrich (Germany). Bovine serum albumin (BSA, cat. no. B9000S) was obtained from New England Biolabs (Germany).

Ampligase DNA ligase (cat. no. A3210K) was purchased from Epicentre. Exonuclease I (cat. no. EN0581), FastAP thermosensitive alkaline phosphatase (cat. no. EF0651), φ29 DNA polymerase (φ-29 Pol, cat. no. EP0094), deoxy nucleoside triphosphates (dNTPs, cat. no. R0192), neutravidin (NA, cat. no. 31050), EDC [1-ethyl-3-(3-dimethylami-nopropyl)carbodiimide], NHS (*N*-hydroxysuccinimide), and Zeba spin desalting columns (7k MWCO, 0.5 mL, cat. no. 89882) were from Thermo Scientific (Germany). DNA sequences specified in Table 1 were obtained from Integrated DNA Technologies (Belgium).

**Table 1:**
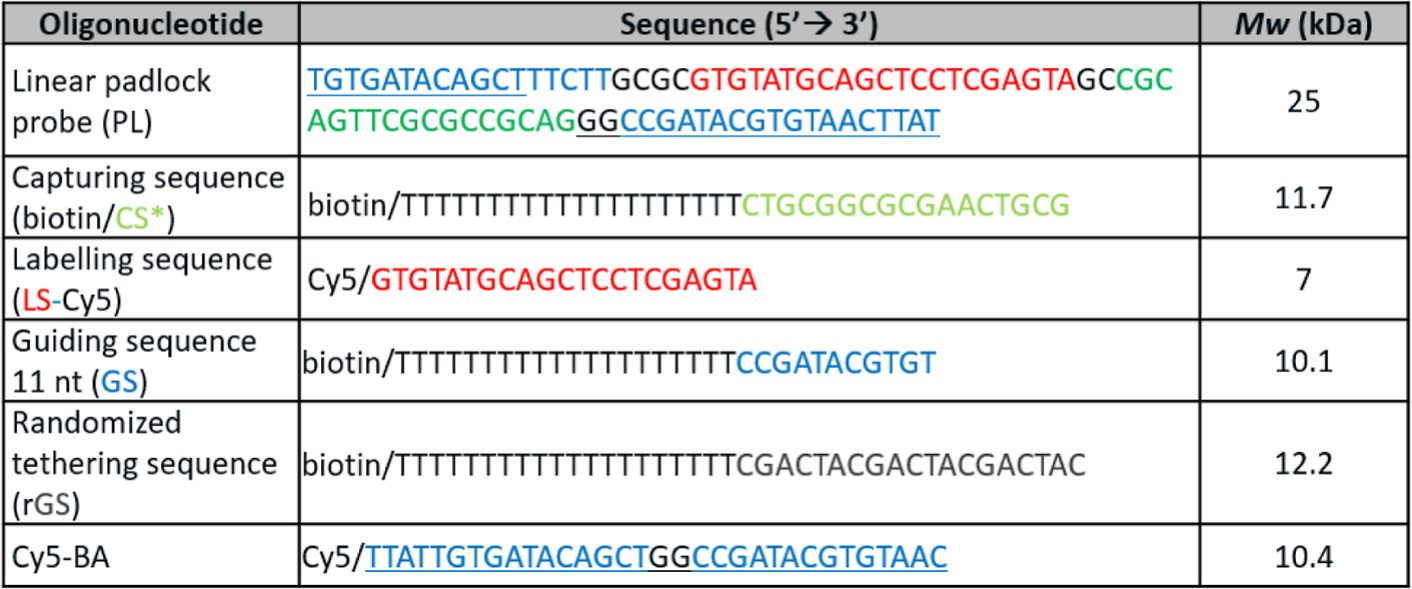
Summary of the Used DNA Sequences for the Experiments with (*) Marking the Complementary Sequences

From sodium acetate [cat. no. S8750 from Sigma-Aldrich (Germany)], a buffer (ACT, 10 mM) was prepared with acetic acid and adjusted to pH 5.55 with NaOH and HCl. The purified monoclonal rat anti-IL6 (clone MQ2-13A5 with product number 14-7069 and MQ2-39C3 with product number 14-7068) was purchased from Invitrogen (Austria) and eBioscience (Austria), while recombinant human IL-6 was obtained from Abcam (Austria, product number ab198571).

### Sensor Chip Preparation

Substrates made of BK7 or LASF9 glass were cleaned for 1 h by the Piranha cleaning procedure (75% of sulfuric acid and 25% of hydrogen peroxide). Then, they were subsequently sonicated with ultrapure water (*R* ≥ 18.2 MΩ/cm^2^), Hellmanex III 1% (v/v), and ethanol, rinsed with pure ethanol, and dried by a stream of pressured air. The cleaned substrates were loaded into a thermal evaporator Auto306 Lab Coater from HHV Ltd (UK), and 2 nm thick chromium layer and 50 nm thick gold layer from MaTeck (Germany) were deposited. The gold-coated slides were incubated overnight in ethanolic solution with dissolved thiols bearing hydroxyl and biotin or carboxyl headgroups (1 mM, mixed at a molar ratio of 1:5). After the mixed thiol self-assembled monolayer (SAM) was formed, the chips were rinsed with pure ethanol and stored under an argon atmosphere in the dark.

### Enzymatic Reactions *Ex Situ*

The linear padlock probe PL was ligated in a total volume of 250 μL. The reaction mixture contained 90 nM concentration of PL, 40 nM concentration of the complementary guiding sequence GS*, 75 units of DNA ligase, and the ligation buffer consisting of 20 mM Tris-HCl, 25 mM KCl, 10 mM MgCl_2_, 0.5 mM nicotineamide adenine dinucleotide (NAD), and 0.01% Triton X-100 dissolved in NFW–BSA (0.2 mg/mL). The ligation reaction was performed at 50 °C on a shaker set to 700 rpm, and it was stopped after 1 h by heating the solution to 85 °C for 15 min. The solution containing the ligated circular padlock probe PL was mixed with 50 units of exonuclease I and 5 units of alkaline phosphatase in the respective buffer [67 mM glycine–KOH, 6.7 mM MgCl_2_, and 1 mM dithiothreitol (DTT)] in a total volume of 500 μL. The reaction was conducted at 37 °C on the shaker set to 700 rpm for 15 min and was terminated by heating to 85 °C for 15 min.

### Growth of DNA Chains by RCA on the Sensor Surface

The thiol biotin-SAM-modified chips were reacted with NA (125 μg/mL in PBST) for 15 min followed by the binding of the biotinylated capture sequence (biotin/CS*; 40 nM in PBST) or a mixture of biotinylated CS* and guiding/randomized guiding GS/rGS sequences (total concentration 40 nM) for 25 min. Finally, the circular padlock probe PL was dissolved in PBST and flowed over the sensor surface for 40 min in order to hybridize with the anchored CS* sequences. Subsequently, the RCA was conducted for 1 h by flowing 100 units of φ-29 Pol in the respective buffer (33 mM Tris-acetate, 10 mM Mg-acetate, 66 mM K-acetate, 0.1% Tween 20, 1 mM DTT, and 100 μM of each dNTP in NFW–BSA) over the surface. The reaction was terminated by rinsing with PBST. The RCA product was labeled by a 15 min reaction with the labeling sequence (LS) oligonucleotide (10 nM in PBST) with the attached Cy5-fluorophore followed by PBST rinsing.

### Preparation of Antibody-DBCO (dAb-DBCO)

The conjugation of anti-IL6 (clone: MQ2-39C3; 0.5 μg/mL in PBS) with the DBCO-NHS ester (0.33 mM) dissolved in DMSO was performed at room temperature for 1 h, according to the manufacture’s procedure. By the addition of Tris-HCl (100 mM, pH 8) for 5 min, the reaction was stopped. In order to remove excess molecules, spin desalting columns were used.

### IL-6 Detection by Immunoassay and RCA

The substrates modified with carboxy-SAM were contacted with sodium acetate buffer with pH 5.55 (ACT) followed by activation *via* freshly prepared EDC/NHS mixture (200 mM/50 mM) for 10 min. The surface was then flushed for 1 min with the ACT buffer, and the capture antibody (cAb; 50 μg/mL) dissolved in the same buffer was reacted with the surface for 20 min. After rinsing with ACT buffer, the remaining active groups were deactivated by ethanolamine (1 M) flowed over the surface for 20 min. Interleukin-6 (IL-6; 47.6 nM) in working buffer was allowed to interact with cAb on the surface for 10 min, and the affinity-captured IL-6 molecules were reacted with the detection antibody labeled with DBCO (dAb-DBCO; 1 μg/mL) for 20 min. The capture labeling sequence (azide-CS*; 40 nM in PBST) for the padlock probe PL was covalently linked dAb by copper-free click chemistry. The RCA reaction was done according to the previous described protocol with PL molar concentration of 40 nM and using the labeling of the RCA product with Cy5-BA sequence (10 nM in PBST).

### AFM Study

The substrates were contacted with NFW and loaded to a JPK NanoWizard III atomic force microscope (AFM, Bruker, Germany). QI mode for imaging was used with 0.1 V force, the extending and retracting time was set to 2.0 ms, the extending and retracting velocity was 100.0 μm/s, and the sampling rate of 200 Hz was used. The AFM tips ScanAsyst-fluid+ (nominal spring constant of 0.7 N/m) were used (Bruker, Germany), and JPKSPM Data Processing software served for the data analysis. Processing included (1) subtracting a linear fit from each line of the image (corrects for height offset in the data line by line) and (2) applying a low pass Gaussian filter (*x* and *y* size of 0.5 px, to denoise the images).

### Optical Setup

**Figure 1:**
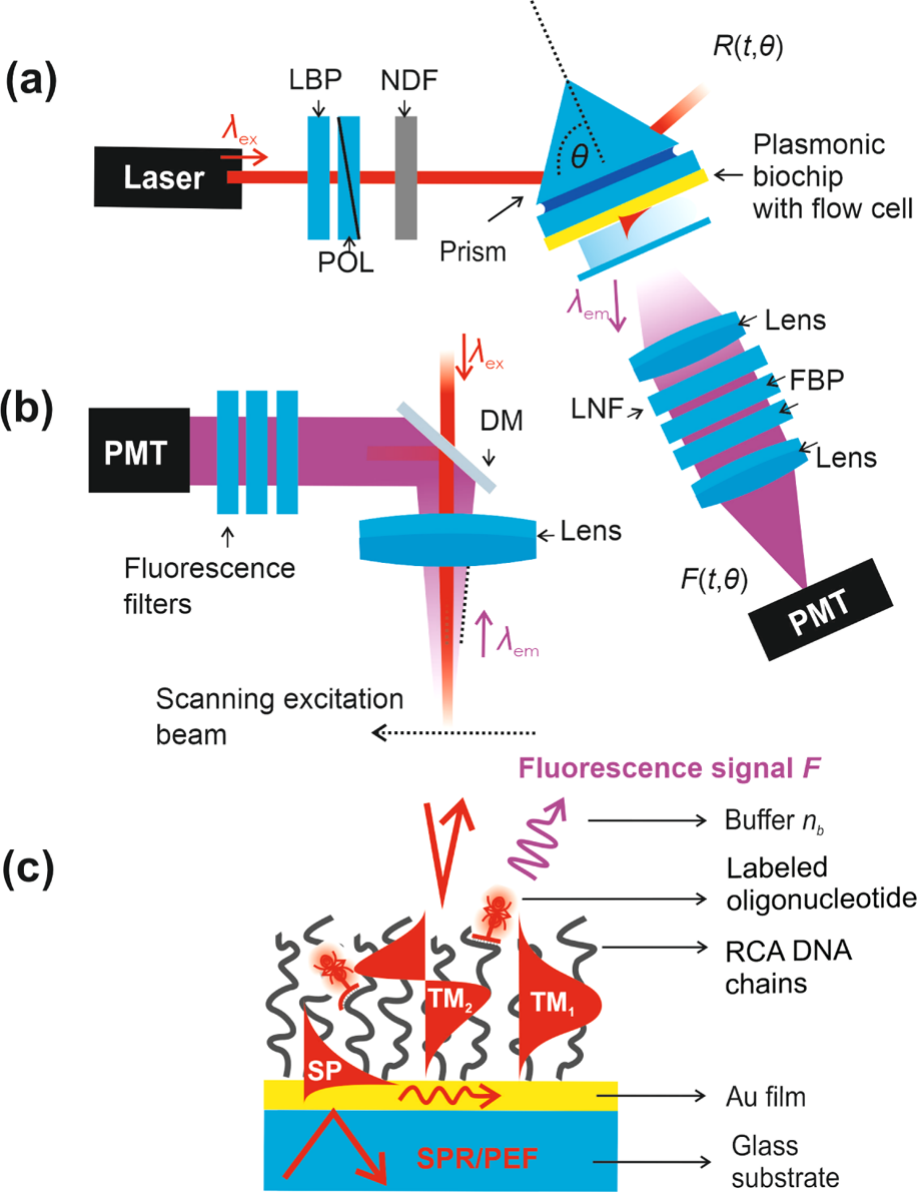
Optical setup employed (a) in the combined SPR and surface PEF spectroscopy and (b) for scanning confocal microscopy measurements with the use of a laser band pass filter (LBP), polarizer (POL), dichroic mirror (DM), neutral density filter (NDF), laser notch filter (LNF), fluorescence band pass filter (FBP), and a photomultiplier (PMT). (c) Schematics of the optically probed biointerface with tethered DNA chains generated by RCA.

The home-built optical instrument with Kretschmann configuration was used for the excitation of SPs and optical waveguide modes (TEM_1,2,..._) on the gold-coated sensor surface (Figure 1). For the optical matching of the sensor chip to a 90° LASF9 glass prism, a refractive index immersion coil (Cargille Laboratories, USA) was used. A HeNe laser beam (λ_ex_ = 632.8 nm) was made travelling through a laser band-pass filter (LBP) and a neutral density filter (NDF) before being launched to the prism. The incident angle θ of the laser beam at λ_ex_ impinging at the sensor surface was controlled by a rotation stage. The excitation beam at λ_ex_ was transversally magnetic (TM) or transversally electric (TE) polarized with a polarizer and a photodiode, which was connected to a lock-in amplifier and was used to measure the reflected beam intensity *R*. A peristaltic pump from Ismatec (Switzerland) transported the liquid sample solutions with a flow rate of 40 μL/min *via* Tygon tubing (inner diameter = 0.25 mm) through the flow-cell with the volume of 10 μL. The reaction chamber forming a flow-cell was sealed by a polydimethylsiloxane gasket and the silica glass substrate with drilled inlet and outlet ports for connecting the tubing. By closing the loop with the tubing, the sample solutions were continuously reintroduced.

Cy5 fluorophores at the sensor surface were excited by the enhanced intensity of SPs at λ_ex_, and the fluorescence light emitted perpendicular to the surface at a red-shifted wavelength of λ_em_ = 670 nm was collected through the transparent flow-cell, collimated by a lens with a focal length of *f* = 50 mm (LB1471 from Thorlabs, UK), and passed through a laser notch filter (LNF, Melles Griot, XNF-632.8-25.0M CVI, USA) and two band pass filters (FBP, Thorlabs, FB670-10 and 670FS10-25 from Andover Corporation Optical Filter, USA), and its intensity *F* was detected by a photomultiplier (H6240-01, from Hamamatsu, Japan) connected to a counter (53131A, *f* = 225 MHz, Agilent Technologies, USA). The reflected beam intensity *R* in % and the fluorescence intensity *F* in counts per seconds (cps) were recorded as a function of time *t* or the angle of incidence θ by using the software Wasplas (developed at Max Planck Institute for Polymer Research in Mainz, Germany).

### SPR Sensor Readout and Data Analysis

The angular reflectivity spectra *R*(θ) were measured for the sensor chip contacted with air and after flowing PBST with the refractive index *n*_b_ through the clamped flow-cell. Then, the angle of incidence θ was fixed below the value where SPR dip minimum occurs and where the slope d*R*/dθ is the steepest. Time-dependent reflectivity measurements *R*(*t*) were used to track changes in SPR. They were quantified in terms of bulk refractive index changes unit (RIU) by calibrating to the response due to the flow of PBST spiked with 1, 2, and 4% (w/w) sucrose yielding refractive indices of *n*_b_ = 1.3344, 1.3359, and 1.3388. In between each reaction, the sensor was rinsed with PBST, and angular reflectivity spectrum *R*(θ) was recorded. By importing the recorded spectra into the software Winspall (developed at Max Planck Institute for Polymer Research in Mainz, Germany), the reflectivity curves *R*(θ) with the SPR dip and the optical waveguide features (manifested as additional dips close the critical angle) were fitted by a Fresnel reflectivity-based model, and the thickness *d*_p_ and refractive index *n*_p_ of the biopolymer layers were determined.

The surface mass density Γ for each biolayer was calculated as Γ = *d*_p_·(*n*_p_ – *n*_b_)/(d*n*/d*c*) in ng/mm^2^, where (d*n*/d*c*) was set to 0.2 mm^3^/mg for proteins and 0.17 mm^3^/mg for DNA. The grafting density σ in nmol/mm^2^ was determined from the surface mass density Γ by taking into account the respective molecular weight *M*_w_. This value can be converted to the number of molecules per area (mm^2^), and the square root of the inverse value results in the average distance between individual chains *D* (see Supporting Information).

## Results and Discussion

**Figure 2:**
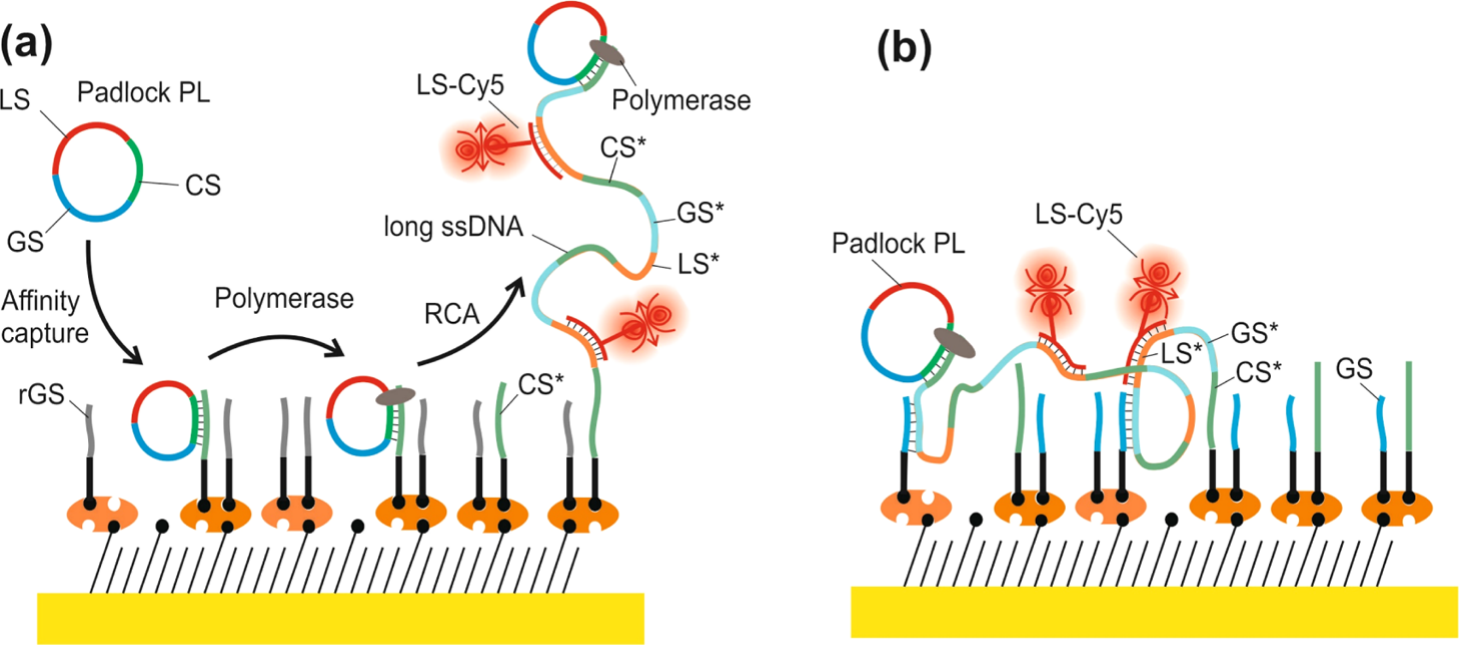
Schematics of the (a) biointerface for the affinity capture of circular padlock probe (PL) *via* the immobilized capture strands (CS*), growth of ssDNA chain (with repeating sequences LS*, GS*, and CS* specified in Table 1), and its reaction with short labeling strands (LS) conjugated with the Cy5 emitter. (b) Visualization of the guiding of RCA-generated long ssDNA chains with the use of mixed ssDNA brush carrying strands with CS* and GS. As a control, a scrambled guiding sequence (rGS) was used.

To investigate the RCA chains tethered on the surface of a plasmonic biosensor, a biointerface architecture that is schematically shown in Figure 2 was used. The gold surface of the sensor chip was modified by a thiol-SAM carrying biotin group, to which NA was anchored. NA served as a linker for subsequent immobilization of capturing ssDNA chains tagged with biotin (biotin/CS*). The CS* sequence was designed to hybridize with a circular padlock probe (PL) that was composed of three segments specified in Table.1 and further referred to as CS, GS, and LS. After the hybridization of the PL with surface-attached CS* strands, isothermal RCA was initiated by the introduction of the φ-29 Pol, leading to the gradual prolongation of the free 3′ end of CS* by incorporating nucleotides (dNTPs). The generated DNA strands carry repeating motifs CS*, GS*, and LS* that are complementary to PL (see Figure 2a). For the used RCA reaction time of 60 min, the previous study^45^ shows that the PL was, on average, rolled over 159 times yielding chains with the contour length of 12.9 kb. The LS* segments carried by long RCA chains were used for the fluorescence detection by affinity reacting with labeled Cy5-LS ssDNA strands. Moreover, we further explored the configuration, where the conformation of the long RCA-generated chains is mediated by affinity interaction with the surface-bearing additional short GS strands (Figure 2b). The surface density of the capture biotin/CS* strands was diluted by co-immobilizing biotin/GS (control randomized) or biotin/rGS strands. The guiding GS moieties were used to anchor the RCA-generated chains *via* the repeating sequences GS* at multiple points and thus restrict its stretching away from the surface outside the evanescent electromagnetic field of SPs.

**Figure 3:**
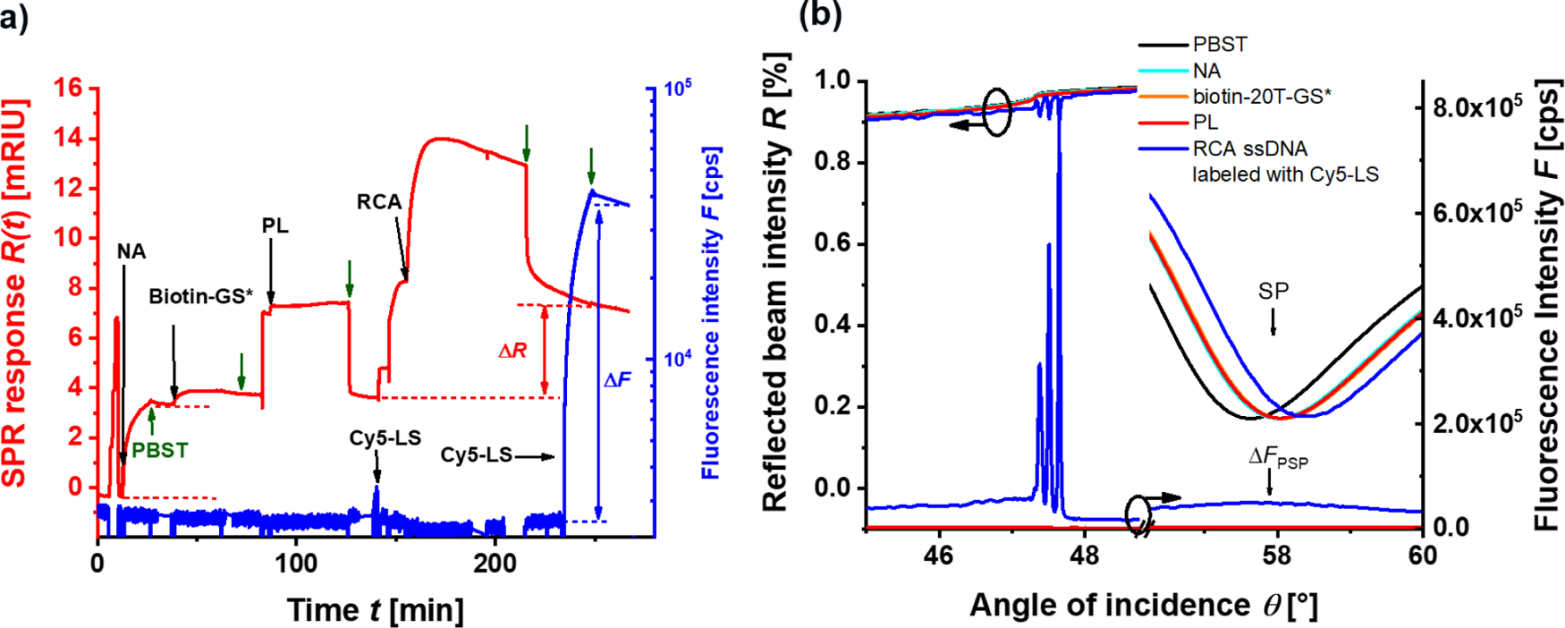
Example of (a) SPR and PEF signal kinetics upon the affinity capture of padlock molecules PL followed by RCA and labeling with Cy5-LS measured at an angle of incidence of θ = 56.7° and (b) measured angular reflectivity and fluorescence scans between the reaction steps.

The assembly of the biointerface, the RCA reaction, and the conformation changes of long ssDNA chains were monitored *via* combined SPR and surface PEF spectroscopy methods. The kinetics measurement of the SPR signal *R*(*t*) captured the variations in the surface mass density associated with changes in the refractive index. The SPR signal was measured at the probing wavelength of λ_ex_ that also served for the fluorescence excitation *via* the enhanced intensity of SPs, and it allowed for recording of the fluorescence intensity *F*(*t*) over time (see Figure 3a). In between the immobilization steps, angular reflectivity *R*(θ) and fluorescence intensity *F*(θ) scans were measured. The reflectivity spectra *R*(θ) exhibited SPR that manifests itself as a dip at an angle of θ ∼ 58° with a width of several degrees. Interestingly, additional sharper dips occurred close to the critical angle θ ∼ 47.4°. They were only present for high density of ssDNA chains that can form a sufficiently thick and dense layer acting as an optical dielectric waveguide. Hence, these resonances are ascribed to optical waveguide modes (marked as TM_1,2,..._ or TE_1,2,..._), and their analysis allowed to determine changes in the biolayer thickness *d*_p_, refractive index *n*_p_, and respective surface mass density Γ. The fluorescence intensity was also recorded as a function of the incident angle *F*(θ) in order to tune the distance, within which the excitation of the conjugated emitters occurs (see Figure 3b). This is possible through the fact that SPs exhibit tightly confined evanescent field reaching about 100 nm from the gold surface, while the dielectric waveguide modes exhibit more delocalized nature and probe longer distances of several micrometers.

Figure 3a shows an example of the sensogram with measured SPR and fluorescence kinetics. The binding of NA to the biotin groups of the thiol-SAM led to an increase of the SPR signal by Δ*R* = 3.72 mRIU. Subsequently, biotin/CS* was attached to the sensor surface *via* the biotin–NA system yielding Δ*R* = 0.44 mRIU. Then, the sensor surface carrying biotin/CS* was reacted with PL dissolved at a concentration of *c* = 40 nM. After the specific hybridization of PL with the CS* that yields Δ*R* = 0.03 mRIU, RCA was initiated by the addition of the polymerase and dNTPs, and the reaction was allowed to run for 1 h. A termination step was applied by flushing the surface with the buffer without the dNTPs and the polymerase enzyme. The respective increase of the SPR response of Δ*R* = 3.60 mRIU is ascribed to the prolongation of CS* and an increase in the surface mass density on the surface Γ. The surface mass density (Γ = 1.93 and 0.06 ng/mm^2^) and the corresponding grafting density (σ = 2.9 × 10^–2^ and 5.0 × 10^–2^ pmol/mm^2^) were obtained for the attachment of NA and the subsequent binding of biotin/CS*, respectively. The presence of the RCA-generated ssDNA strands was further confirmed by the specific affinity binding of Cy5-LS, yielding a strong fluorescence intensity increase by Δ*F* = 4.73 × 10^4^ cps that was observed only after the RCA reaction (and no signal was measured upon the same reaction prior to the RCA). The grafting density of the RCA-prolonged ssDNA chains was controlled between σ = 3.1 × 10^–3^ and 3.6 × 10^–6^ pmol/mm^2^ by the concentration of PL in a solution that was reacted with CS* on the sensor surface (see Figure S1 in Supporting Information, Section S2). It was determined by combined SPR and PEF methods, and a dependence of PL concentration *c* and the average distance between the anchor points *D* was established.

### Conformation Changes of ssDNA Chains

**Figure 4:**
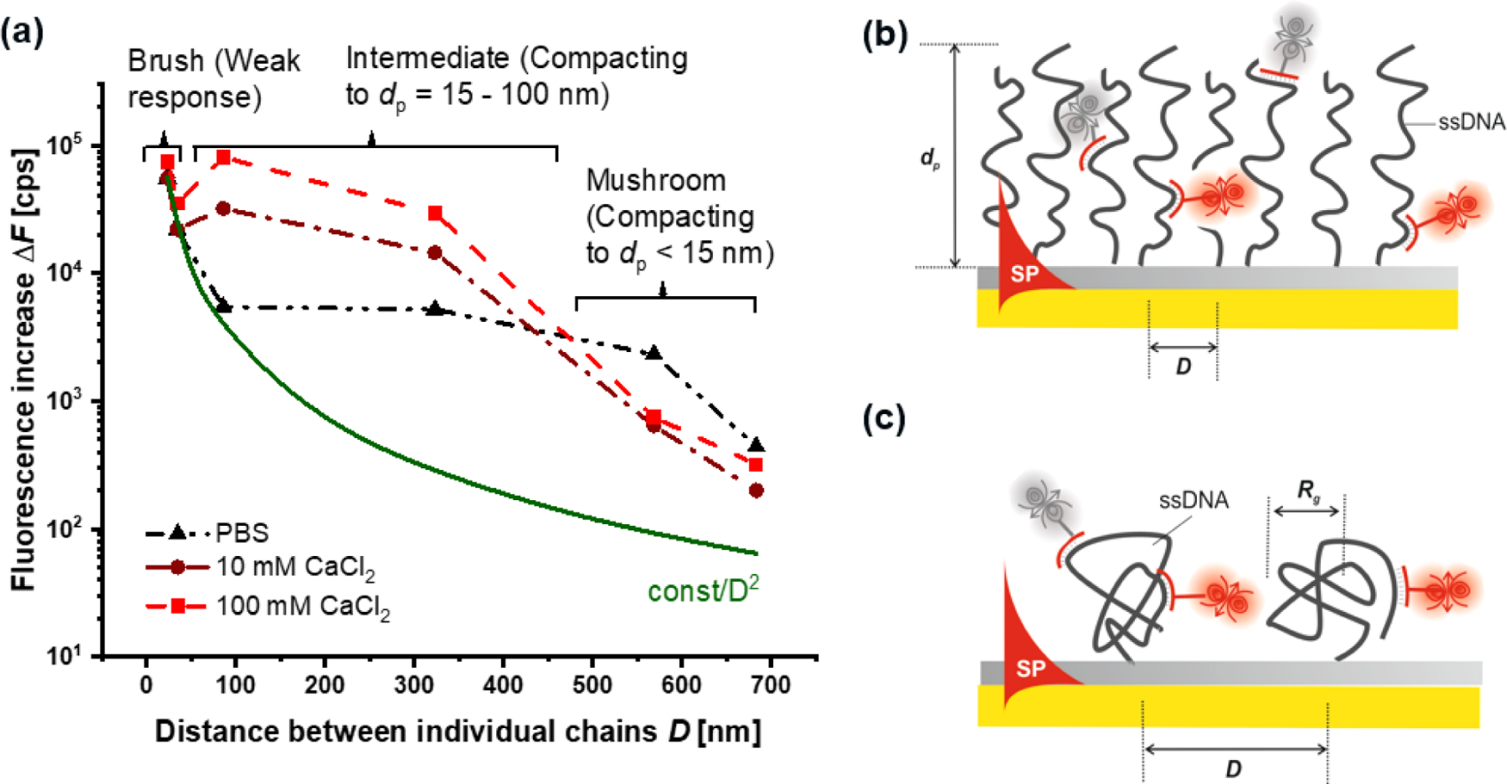
(a) Dependence of the measured fluorescence signal for excitation *via* the resonantly excited SPs on the sensor surface for varied average distance between ssDNA chains *D* (dashed lines between measured data are guides for eye) and schematics of ssDNA chains taking (b) dense brush and (c) sparse random coil conformation.

A series of RCA experiments was performed with varied average distance *D* between the anchoring points, from which the ssDNA chains were grown. This distance range was determined between *D* = 20 and 700 nm, and after each RCA reaction, the ssDNA chains (with a contour length > 10 μm) carrying repeating GS*, CS*, and LS* motifs were reacted with short complementary Cy5-LS strands. Then, the fluorescence intensity response Δ*F* was measured upon probing the sensor surface with the confined field of SPs decaying to a short distance of about 100 nm from the sensor surface. These response values were determined from the angular fluorescence scans *F*(θ) as an increase in the fluorescence intensity with respect to the background at the SPR angle. The obtained dependence of fluorescence response on the average distance between anchoring points Δ*F*(*D*) is presented in Figure 4a (showed as circles, squares, and triangles connected with dashed and dotted lines), and apparently, these data strongly deviate from the assumption that the fluorescence intensity is simply proportional to the surface density of anchoring points (showed as a solid green line representing a function of const/*D*^2^). This deviation suggests that the conformation of the ssDNA chains changes when diluting the anchoring points, and thus the amount of fluorophores per attached chain that can be excited within the close proximity to the metal surface (<100 nm) is altered.

First, let us discuss the dependence Δ*F*(*D*) measured in physiological buffer (PBS, black triangles). It shows a rapid decrease of the fluorescence intensity when increasing *D* at short distances below 100 nm. Then, the fluorescence response Δ*F* weakly changes with distance *D* for the sparser arranged anchoring points at *D* = 100–300 nm, and finally, it again decreases with *D* for long average distances between the anchoring points >300 nm. This behavior can be ascribed to the transition from the (polyelectrolyte) brush architecture taken by densely packed chains (*D* < 100 nm) to random coil mushroom conformation at low grafting densities (*D* > 300 nm). The polyelectrolyte brush architecture is established due to repulsive interactions between the negatively charged backbone of the ssDNA in the high-density regime (illustrated in Figure 4b), forcing the chains to stretch away from the sensor surface. ssDNA then forms a layer with a thickness of several μm, which is far thicker than the probing distance of SP waves, and thus a only small fraction of fluorophore emitters decorating the chains in the vicinity of the gold surface are excited. By increasing the distance *D* between the ssDNA chains from *D* = 23 to 83 nm, the fluorescence intensity drops down, and it suggests that the chains retain their highly stretched conformation as the amount of excited fluorophores scales with the anchoring density. ssDNA can be expected to adopt a mushroom conformation (illustrated in Figure 4c) when the grafted chains cease interacting with their neighbors at distances above their random coil size. In this regime, the fluorescence signal Δ*F* would be decreasing with *D*, as can be seen for the average distances between the anchoring points >300 nm. The coil size of the isolated RCA ssDNA chains can be estimated as *R*_g_ = 179 nm by using the Flory–Huggins model for a good solvent with ***v*** = 0.588, which agrees with these observations. The region where the fluorescence response weakly depends on the distance occurring at *D* = 100–300 nm thus can be ascribed to the transition between these two regimes, when the chains are allowed to partially back fill the space between the tethering points and thus bring chain segments carrying the fluorophore emitters from far distances to the proximity to the metal probed by the confined SP field.

As the stretching of the ssDNA chains appears to strongly affect the measured fluorescence response Δ*F*, we explored the effect of exposing the RCA-generated ssDNA chains to calcium ions Ca^2+^. The negatively charged ssDNA Coulombically interacts with the positively charged Ca^2+^ ions leading to partial screening of the repulsion forces, and also a specific cross-linking effect between the chains can occur. The ssDNA layer can then partially collapse, resulting in the pulling of a fraction of the fluorophores from the upper part of ssDNA chains toward the SP-probed volume, which manifests as an increase in the fluorescence intensity Δ*F*. As Figure 4a shows for 100 mM Ca^2+^, this effect is weakly pronounced for dense ssDNA polyelectrolyte brushes formed by strong repulsion between the neighboring chains. Then, only a small increase of the fluorescence intensity by a factor of 1.35 at *D* = 23 nm and 1.59 at *D* = 34 nm is observed. The exposure to Ca^2+^ at the same concentration showed a substantially stronger impact in the transition region, where the decreasing trend of fluorescence response with *D* was even reversed and Δ*F* was enhanced by a factor of 15 at *D* = 85 nm and 5.7 for *D* = 323 nm with respect to values measured in PBS. This effect may be explained by the possible cross-linking effect of Ca^2+^ and the weakened repulsion between the chains, which may drive the back-filling of the SP-probed volume through compacting the ssDNA strands. Interestingly, an opposite optical response was observed for long distances *D*, where the ssDNA chains take the mushroom conformation. A decrease in the fluorescence response Δ*F* by a factor of 3.6 at *D* = 568 nm and 2.2 at *D* = 700 nm was measured with respect to PBS. This effect can be ascribed to fluorescence quenching due to the pulling of the chains to the very close proximity of the metal^46,47^ to distances *d*_p_ < 15 nm. Then, the Ca^2+^ ions possibly decrease the size of the ssDNA random coil far below 100 nm by the screening of repulsion and cross-linking between different segments of individual chains. In general, lowering the Ca^2+^ concentration leads to similar, but less pronounced, effects.

### Guiding of ssDNA on the Sensor Surface

When applied for the amplification of assay with a plasmonic biosensor readout, long ssDNA chains generated by RCA requires matching their conformation with respect to the volume probed with confined SP field. In order to keep the chains within the optimum distance from the metallic surface, additional mechanism based on multipoint attachment was investigated by using the biointerface architecture shown in Figure 2b. This approach was implemented by using GSs constructed of 11 and 32 nucleotides complementary to GS*, a 20 nucleotide thymine spacer, and a biotin group to bind to the NA-coated sensor surface. It is worth noting that the GS with 32 nucleotides length quenched the RCA on the sensor surface, which was ascribed to a too strong interaction with the RCA product. Therefore, the shorter 11-mer GS version with presumably weaker affinity was used. In the control experiment, a rGS was used, and the fluorescence signal increase Δ*F* was also measured for the PL probe directly labeled with Cy5.

**Figure 5:**
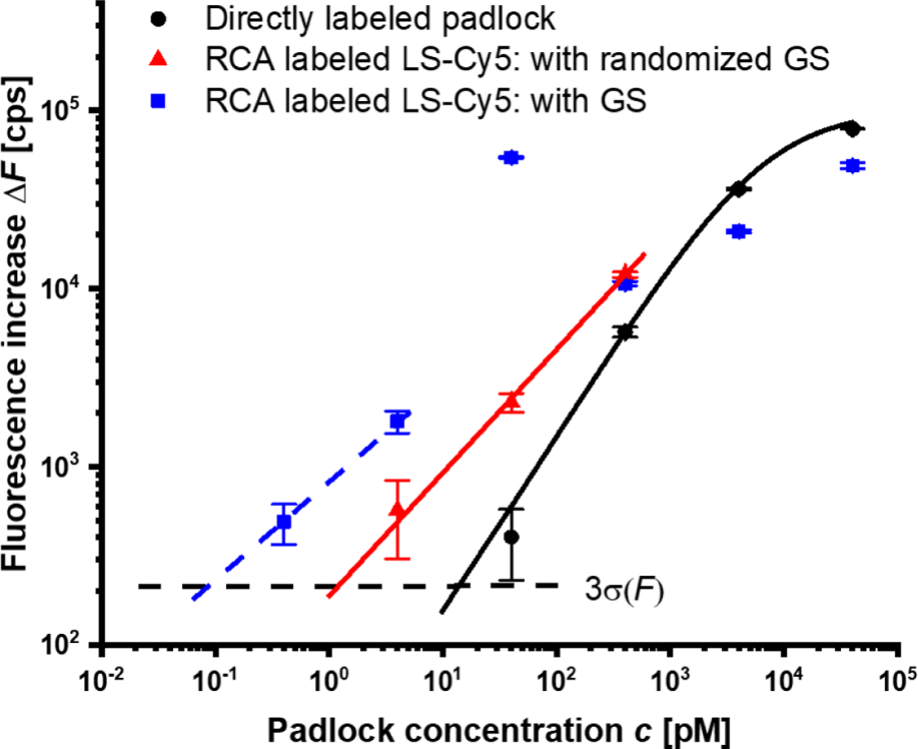
Comparison of the calibration curves for the PEF biosensor for an RCA assay with direct labeling of padlock molecules, RCA on the biointerface carrying randomized rGS strands, and RCA on the biointerface with short complementary GS strands. The measurements were performed after exposure of ssDNA to high ionic strength solution and rinsing the surface with a running buffer. The black line represents a fit with the Langmuir model function, the red curve represents the linear fit, and the blue dashed line is a guide for eyes. Error bars were determined as the standard deviation of readout noise.

Figure 5 shows the established dependence of the surface PEF signal Δ*F* on the concentration of the padlock PL. This signal was measured after the surface carrying the biointerface with the mixed GS and CS* sequences was reacted with a PL that represents a model analyte and was dissolved in the working buffer. The figure compares the obtained calibration curves for the directly labeled PL with Cy5-LS* for the RCA chains generated at the points where the PL was affinity-captured followed by reacting with Cy5-LS and for the same experiment with RCA amplification on the surface with the GS sequences replaced by the randomized control rGS. The data points were extracted from the maximum of the fluorescence scans *F*(θ) measured after ssDNA was exposed to high ionic strength Ca^2+^ followed by rinsing with a working buffer. The chip-to-chip reproducibility of the measured fluorescence signal for the same concentration of PL was determined as the standard deviation σ(Δ*F*) divided by average Δ*F* yielding 10%.

The curve for the directly labeled PL with Cy5-LS* shows a dependence that can be fitted with the Langmuir isotherm saturating at PL concentrations above 1 μM. The LOD was determined as the concentration at which the fitted function crosses the 3 times standard deviation of the baseline noise 3 × σ(*F*) = 200 cps, and it yields 13 pM.

For the fluorescence signal enhanced by RCA, the calibration curve shows non-Langmuir behavior, and at nM concentrations, the measured fluorescence intensity Δ*F* is below the values obtained for directly labeled padlock PL. However, when decreasing the PL concentration below nM, this RCA dependence crosses over that for directly labeled PL, and the enhanced response provides LOD that can be estimated close to 0.1 pM, yielding an improved factor of about 10^2^. The calibration curve exhibits the maximum fluorescence signal Δ*F* close to the PL concentration of 40 pM, which corresponds to the transition region from brush to random coil conformation (as observed previously in the study of the fluorescence intensity Δ*F* depending on the average distance between the chains *D* that is presented in Figure 3). The contribution of the confinement of ssDNA on the sensor surface carrying 11-mer GS sequences was tested by running the same RCA experiment on a surface with rGS sequences that do not bind to the strand complementary to PL. Then, the fluorescence response Δ*F* was weaker and led to an only 13-fold improved LOD with respect to directly labeled PL. Based on the previous study,^45^ it can be estimated that RCA strands are formed by rolling the PL by about 159 times for the used conditions. This value corresponds to the maximum increase in the number of anchored fluorophores per affinity-captured PL molecules with respect to the direct labeling, and the achieved maximum enhancement in LOD approaches this factor. This result indicates that the designed biointerface enabled minimizing the effect of possible quenching by the metal surface, the self-quenching effect by coupling neighboring emitters, and the conformation-limited accessibility of LS segments for the binding of complementary Cy5-LS*.

### Fluorescence Microscopy Observations of the Sensor Chip

In the above SPR and PEF experiments, the fluorescence response was averaged over the area of several mm^2^, and thus it originates from ensembles of affinity-captured molecules. Therefore, they were complemented by fluorescence microscopy observations of the sensor chip after the RCA assay. The study was conducted with molar concentrations of PL ranging from *c* = 40 fM to 40 pM (and control experiment with *c* = 0) by using the same RCA reaction protocol. The generated ssDNA was subsequently labeled with Cy5-LS (as shown in Figure 2b). The fluorescence intensity of the RCA product was increased compared to the dry sensor chip by contacting it with the buffer and sealing the surface by a glass coverslip (which can be ascribed to the reduced effect of quenching occurring on the dry interface).

**Figure 6:**
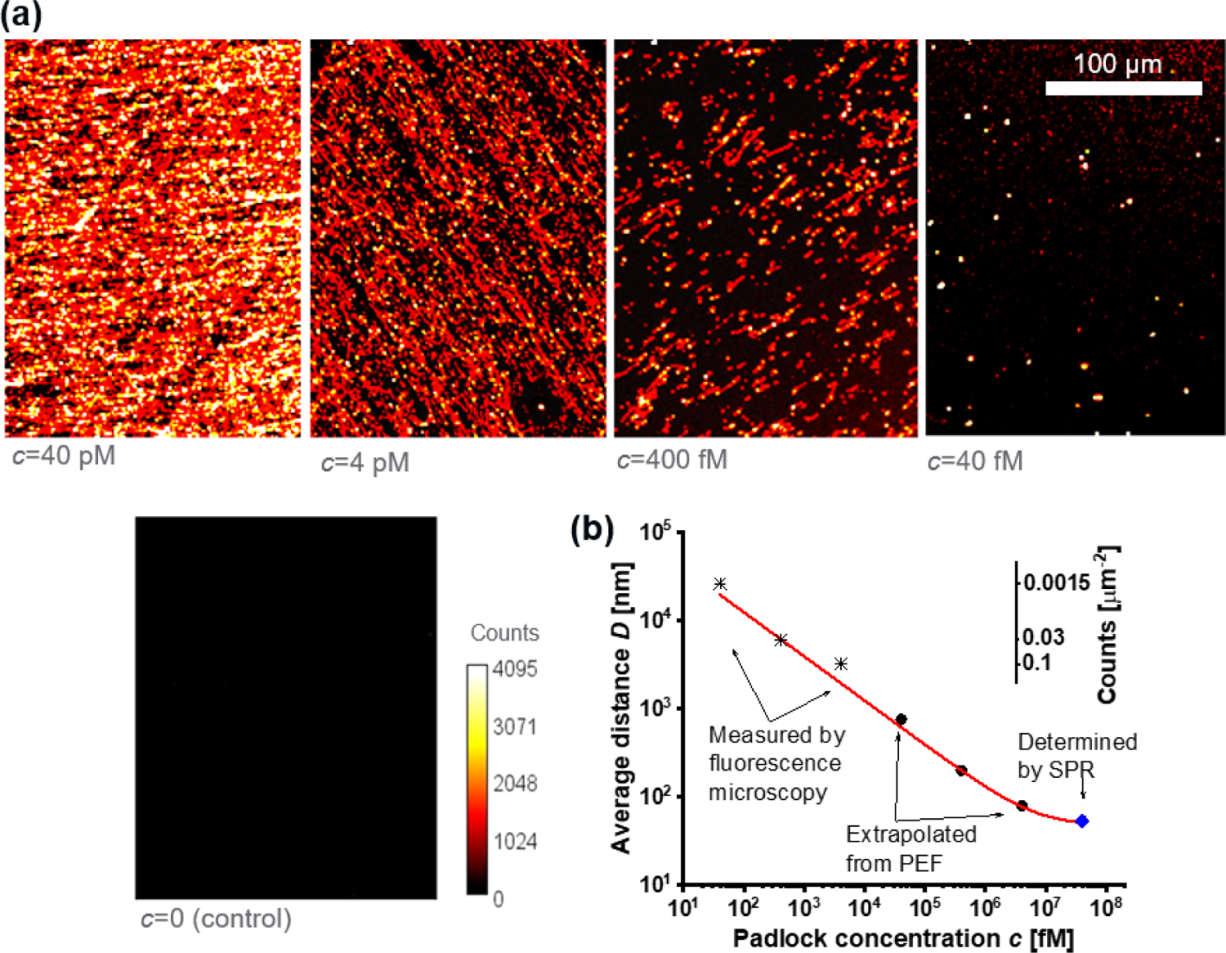
(a) Fluorescence microscopy observation of the fluorescence signal from the sensor chip surface carrying RCA chains labeled with LS-Cy5 that were generated with the use of padlock affinity-captured from a solution with the padlock concentration of *c* = 40 fM to 40 pM and *c* = 0 as the control experiment. (b) Comparison of the average distance between the affinity-captured padlock molecules on the surface depending on its concentration in the solution contacted with the sensor surface.

The acquired images are presented in Figure 6a and for high molar PL concentrations, they reveal a dense structure of entangled chains. However, well-separated bright spots become apparent for sub-pM concentrations of PL when the grafting density is reduced, and these spots can be ascribed to individual ssDNA produced by RCA. The average distance *D* between them was determined by counting these spots (see details in the Supporting Information). The results are correlated with the molar concentration of PL and compared to the data points acquired from the SPR and PEF (Figure 6b). These data follow the same trend and support the conclusion that the bright spots in the fluorescence images correspond to the locations where individual PL molecules are bound and where subsequently the long ssDNA chains were synthesized. The individually bound DNA chains were also examined with AFM as can be seen in Figure S5 for the substrate carrying the GS and the control rGS sequences. The grafted RCA-generated chains were probed in water by an AFM tip with a spring constant of 0.7 N/m (more details provided in Supporting Information, Section S6). On the reference surface with scrambled rGS sequences, they manifested themselves as spots with a height of about 30 nm and lateral size above 1 μm. Let us note that the lateral size is substantially higher than the estimated gyration radius of the ssDNA chain coil, which can be attributed to the potential detachment and partial aggregation on the surface. On the surface with the GS sequences, similar height of ssDNA chains was observed and the lateral size was substantially decreased to about 100 nm as expected for individual chain coils.

### Implementation to Immunoassays

**Figure 7:**
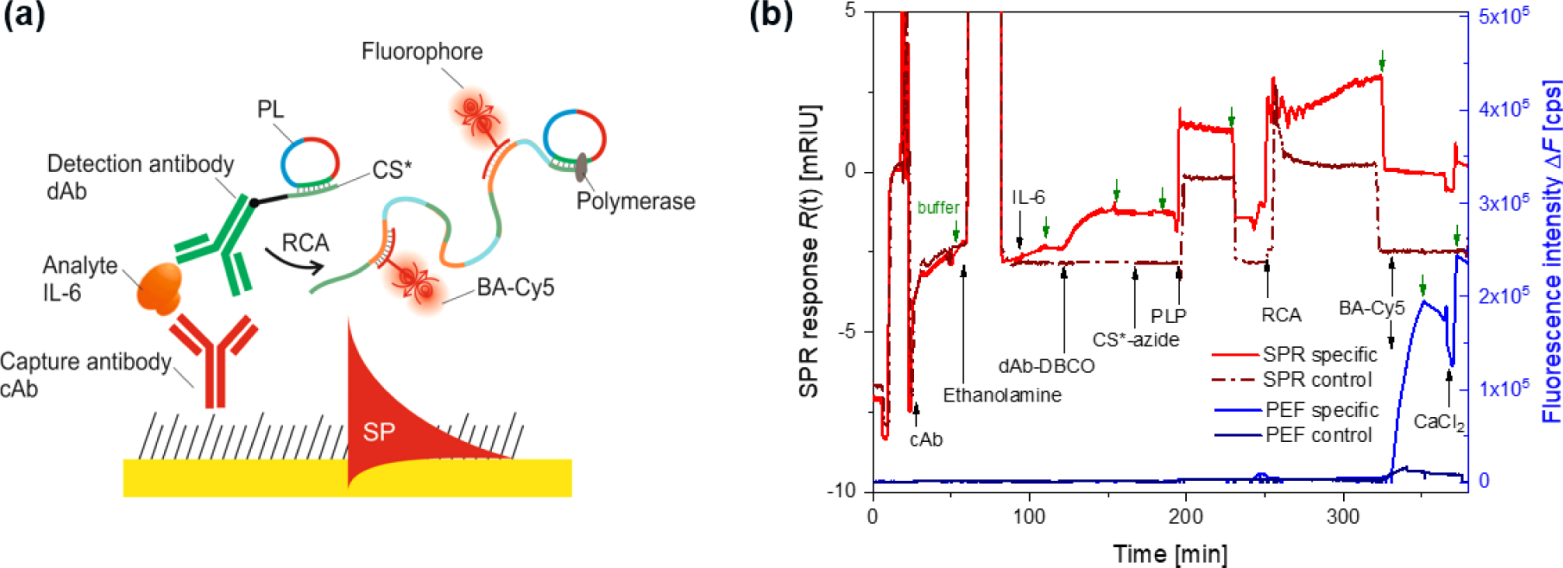
(a) Schematics of the RCA implementation to sandwich immunoassay on a metallic surface with mixed thiol SAM. (b) SPR and PEF readout of the formation of the biointerface and sandwich assay with the concentration of target IL6 analyte of *c* = 47.6 nM (specific) and *c* = 0 (control) followed by RCA and reaction with complementary fluorophore-tagged BA labeling strands.

The approach of imaging of individual RCA chains grown on a surface can provide a versatile route to a digital readout of assays in a partitioning-free manner.^48,49^ For instance, such a detection format can be realized in combination with the sandwich immunoassay by using detection antibody conjugated with a short oligonucleotide tag.^49^ As illustrated in Figure 7, sandwich immunoassay with RCA is herein demonstrated by using a gold plasmonic sensor surface that is modified with mixed thiol SAM-carrying carboxyl groups for the covalent immobilization of cAb. This cAb was specific to the chosen target analyte—interleukin 6 (IL-6, dissolved to a concentration of *c* = 47.6 nM and control *c* = 0)—that was reacted with the sensor surface for 10 min followed by the coupling of detection antibody dAb for 20 min. The dAb was conjugated with DBCO in order to subsequently bind by copper-free click chemistry the ssDNA strand CS* *via* its azide end group. Afterward, PL was hybridized with CS*, and RCA reaction with the SPR and fluorescence readout was carried out by using the same protocol as described previously (for a PL concentration of 40 nM). The analysis time (excluding the immobilization of cAb) was about 250 min, which is substantially longer than that for similar assays with directly labeled dAb. However it should be noted that it can be efficiently shortened by reducing the number of assay steps (*e.g.*, prereacting dAb-DBCO with azide-CS* hybridized with PL and by combining the RCA with labeling reaction).

Figure 7b shows the acquired sensorgram of the SPR and fluorescence readout kinetics for the described immunoassay. It shows that the specific capture of IL-6 by the cAb leads to an increase in the SPR signal by Δ*R* = 0.36 mRIU. The subsequent affinity binding of dAb to IL-6 showed the stronger response of Δ*R* = 1.16 mRIU due to its higher molecular weight, while the response on the control surface was not measurable. The RCA was performed after the coupling of CS* and PL molecules, leading to the increase in the SPR signal on the specific surface by Δ*R* = 1.52 mRIU (and not measurable on the control surface). This change is associated with the prolongation of CS* sequences anchored to dAb as is proven by the strong increase of the fluorescence intensity after labeling with BA-Cy5 (let us note that this labeling sequence was chosen based on the previous work due to its ability to partially cross-link the RCA chains^45^). The specific plasmonically enhanced fluorescence response of Δ*F* = 1.88 × 10^5^ cps is comparable to that measured in the experiments with model PL experiments (see Figure 3). However, a substantially increased background signal of Δ*F* = 1.29 × 10^4^ cps is observed on the control surface, which is attributed to the effect of unspecific sorption of dAb on the surface (even it is low and was not measurable by SPR). These data show that RCA allows for achieving a very high amplification yield (here by about 2 orders of magnitude), but the utilization of the antifouling biointerface is of utmost importance in order to fully harness its potential in the presented sandwich immunoassays. The avoiding of the amplification of the background signal (occurring due to unspecific sorption of assay constituents and abundant molecules present in realistic complex samples) can be potentially achieved with currently developed advanced polymer brush architectures^50−52^ that are documented to provide resistance to unspecific sorption at levels superior to thiol SAM representing the gold standard in the currently used plasmonic biosensors.

## Conclusions

This work concerns tailoring of RCA to efficiently serve in ultrasensitive plasmonic biosensors through confinement of long ssDNA chains in the evanescent field of SPs. The conformation of generated ssDNA and the strength of the respectively amplified response was investigated by a combined setup of SPR and PEF. A transition from a dense polyelectrolyte brush architecture to sparsely distributed random coil conformations of the grafted ssDNA was observed as a function of surface density of the anchoring points. The presented approach for guiding of long RCA-generated chains along the biointerface with weak affinity tethering ssDNA sequences holds potential to improve the performance characteristics of various assays, where short oligonucleotides are used as a tag on a solid surface of physico-chemical transducer (as shown for sandwich immunoassay). The reported investigations show that the fluorescence response can be enhanced by more than 2 orders of magnitude with respect to direct labeling approach. This factor is close to the maximum value (that is associated with repeating number of padlock sequences), which confirms the proper folding at the optimum distance from the gold surface. The LOD at high fM was achieved for the PEF readout, and further improvement to low fM range can be reached when microscopy-based imaging in conjunction with counting of captured molecules is performed. The combined RCA and plasmonic enhancement of the fluorescence response can pave the way to a more accurate readout and possible shortening of the RCA reaction time yielding faster detection. In addition, it offers an opportunity to achieve signal-to-noise ratios suitable for screening of larger surface areas that can accommodate arrays of spots for the parallel analysis of multiple analytes, as well as the implementation of simpler optics that do not require using high-end microscopes.^53^

## Supporting Information Available

Overview of bioassays with RCA amplification probed on a solid surface of various types of physico-chemical transducers, data on the calibration of RCA chains anchoring density, information on the effect of quenching of RCA depending on the length of GSs, fluorescence microscopy imaging data processing, gel electrophoresis, and AFM observation of RCA chains (PDF)

## Abbreviations

SPR: surface plasmon resonance
PEF: surface plasmon-enhanced fluorescence
RCA: rolling circle amplification
ssDNA: single-stranded deoxyribonucleic acid
PL: padlock probe
