# Supplementary material for: Rolling Circle Amplification Tailored for Plasmonic Biosensors: From Ensemble to Single-Molecule Detection
Source: ACS Appl Mater Interfaces. 2022 Nov 29;14(49):55017–27. doi: 10.1021/acsami.2c14500 (PMC9756284; doi:10.1021/acsami.2c14500)
Supplement: Supplementary file 1 — am2c14500_si_001.pdf [file am2c14500_si_001.pdf]

Supporting Information to:

Rolling Circle Amplification Tailored for Plasmonic Biosensors: from Ensemble to Single Molecule Detection

Katharina Schmidt,[†,‡] Simone Hageneder,[†] Bernadette Lechner,[†,‡] Barbara Zbiral[#], Stefan Fossati,[†] Yasaman Ahmadi,[§] Maria Minunni,[⊥] Jose Luis Toca-Herrera[#], Erik Reimhult,[#] Ivan Barisic,[§] Jakub Dostalek[†‖*]

† Biosensor Technologies, AIT-Austrian Institute of Technology GmbH, Konrad-Lorenz-Straße 24, 3430 Tulln an der Donau, Austria

‡ CEST Competence Center for Electrochemical Surface Technologies, 3430 Tulln an der Donau, Austria

§ Molecular Diagnostics, Health & Environment, AIT Austrian Institute of Technology GmbH, 1210 Vienna, Austria

Institute for Biologically Inspired Materials, Department of Nanobiotechnology, University of Natural Resources and Life Sciences Vienna (BOKU), Vienna 1190, Austria

⊥ Department of Chemistry "Ugo Schiff", University of Florence, via della Lastruccia 3-13, Sesto Fiorentino, 50019 Firenze, Italy

‖ FZU-Institute of Physics, Czech Academy of Sciences, Na Slovance 2, Prague 182 21, Czech Republic

1. Overview of assays utilizing RCA-based amplification of output signal

RCA increasingly serves as an amplification method in assays that rely on the affinity capture of target analyte species on a solid surface of a physico-chemical transducer. Table S1 provides selected examples of the optical, microbalance, magnetic, and electrochemical methods employed for the RCA-amplified assay readout with information on the medical diagnostics related application and achieved analytical performance (in terms of limit of detection - LOD).

Table S1. Examples of RCA-based assays with information on the transducing mechanism, application, and achieved limit of detection.

Method	Target analyte & Applications	Limit of detection	Reference
Fluorescence microscopy	synthetic DNA from *Pseudomonas aeruginosa*; Automation in diagnostic laboratories	1 aM	1
Fluorescence scanner	Multiplex detection systems for nucleic acid	0.1 nM	2
Fluorescence microscopy	Salmonella; diagnostics, food contamination, environmental monitoring	100 fM	3
Surface plasmon resonance	Detection of vascular endothelial growth factor; clinical applications	2.22 fM	4
Surface plasmon resonance	*Mycobacterium Tuberculosis*; Multiplex mutation detection, clinical samples	5 pM	5
Surface plasmon resonance, Quartz crystal microbalance	Human α-thrombin; biological research & clinical diagnostics	0.78 aM	6
Quartz crystal microbalance	Hepatitis B virus; clinical applications	10^4 copies/mL	7

Magnetic nanobead-based	Synthetic DNA from *Vibrio cholerae*; rapid diagnostics	5 pM	8
Impedance spectroscopy	Adenosine; biomedical applications	320 pM	9
Electrochemical	exosomal microRNAs; medical diagnostics	2.75 fM	10
Electrochemical	Small molecule-proteins; diagnostics	0.4 pM	11

2. Calibration of the surface density of anchoring points

The circular padlock probe PL was prepared according to the description given in 'Methods' section of the manuscript. After the ligation and exonuclease reaction, the obtained circular PL was diluted to a given concentration c_{PL} and ex-situ labelled by reacting with Cy5-LS* for 10 minutes before it was contacted with the pre-functionalized sensor surface carrying biotin-CS* probes. Solutions with increasing molar concentration of PL/Cy5-LS* complex (c = 4 pM to 40 nM) were flowed over the sensor chip surface and the fluorescence signal $\Delta F(t)$ was recorded by the PEF method. The fluorescence response ΔF was determined after the rinsing with working buffer (difference in the fluorescence intensity before and after the affinity binding) as the function of the molar concentration c and, as shown in Figure S1a, it saturates at c > 10 nM and exhibits Langmuir isotherm behavior. For the highest concentration of c = 40 nM, the surface mass density of the affinity captured padlock PL of $\Delta\Gamma$ = 0.18 ng/mm^2 was determined (as it induced a detectable shift in SPR) and it was related to the respective fluorescence response ΔF . By such determined conversion factor $\Delta F/\Delta\Gamma$, the surface mass density increase was extrapolated for all other lower concentrations c and average distance between the tethering points was calculated based on the known PL molecular weight as $D = \sqrt{M_W \cdot NA/\Delta\Gamma}$, (see Figure S1b).

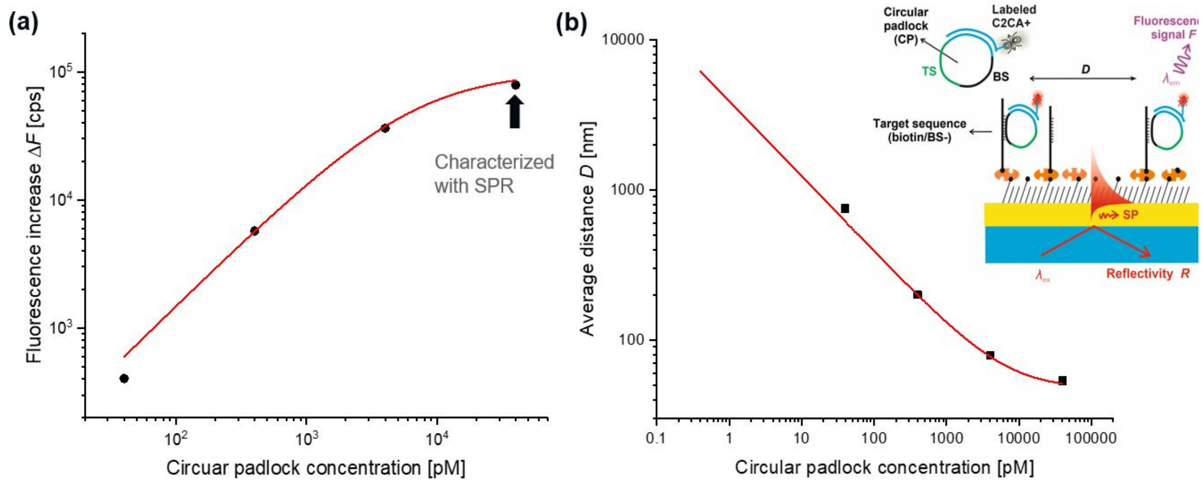

Figure S1. a) Fluorescence intensity ΔF measured for pre-labelled padlock probe with molar concentrations ranging from c_{PL} = 40 pM to 40 nM correlated , b) relation between the molar concentration of the padlock probe c_{PL} reacted with the sensor surface with the calculated average distance between the tethering points D.

3. Affinity guiding of ssDNA chains on the surface

The guiding of the RCA-generated long ssDNA strands by affinity interaction of repeating chain segments with the sensor surface was first tested with a guiding sequence constructed of a biotin group, a 20-nucleotide thymine spacer and 32 sequence of GS strands. Interestingly, the RCA was then quenched and therefore the guiding sequence GS was shortened to 11 nucleotides (see Table 1 in the manuscript) in order to allow for weaker reversible attachment of RCA product. Then, the RCA process was not impaired and pronounced shift of SPR in the angular reflectivity curve occurs after the reaction (as can be seen in Figure S2a.). Additionally, two sharp dips at the location of the critical angle occur in the reflectivity spectrum $R(\theta)$, which indicate the production of a thick ssDNA layer supporting two dielectric waveguide modes. The surface mass density of the RCA chains was then obtained by fitting $R(\theta)$ with a Fresnel multilayer model. The determined refractive index n_p and

thickness d_p translates to high increase in surface mass density of $\Delta\Gamma$ = 98.27 ng/mm^2 and was accompanied with strong fluorescence response ΔF = 5.94·10^4 cps was extracted from the angular fluorescence scans $F(\theta)$ for the excitation via surface plasmon waves.

Exposing the ssDNA to solutions with Ca^{2+} (c = 10 mM) led additionally enhanced fluorescence signal ΔF in the low-density regime for D = 320 nm by a factor of 10.48, as shown in Figure S2b (red bars) on the interface carrying the 11-mer GS sequences. In the control experiment with randomized rGS strands, this strong enhancement is not observed. For the denser structure with D < 100 nm, the repulsion between tightly packed chains probably prevented the affinity binding of the RCA-strands to the guiding sequences GS, hence no enhancement effect was measured.

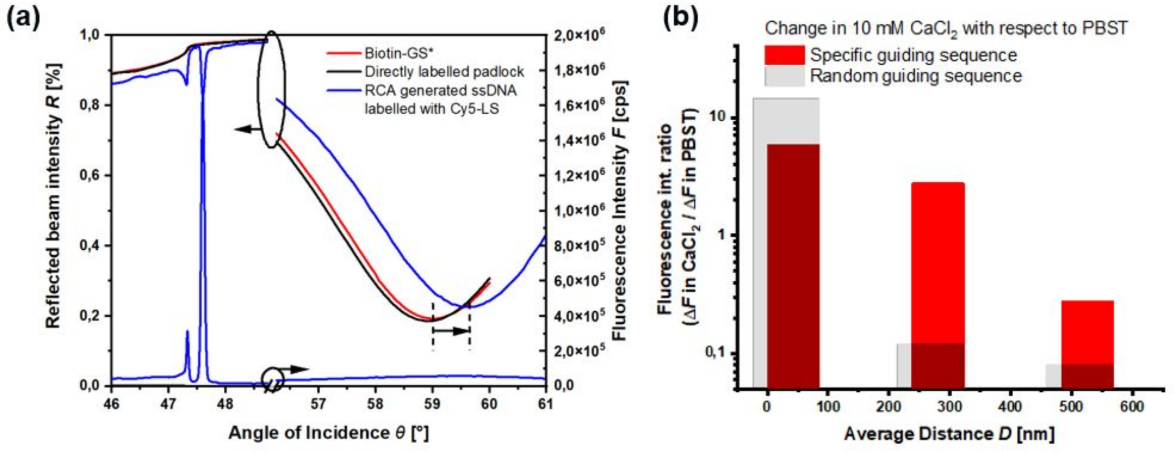

Figure S2. a) Angular reflectivity $R(\theta)$ and fluorescence scans $F(\theta)$ of a sensor surface reacted first with biotin-CS* and biotin-GS in a 1:1 ratio (red), subsequently binding of directly labeled PL with Cy5-LS* (black) and after one hour of RCA reaction, ssDNA reacted to Cy5-LS (blue), b) change of the fluorescence signal of Cy5-LS labeled ssDNA represented as ratio of ΔF when the strands are exposed to $CaCl_2$ and in PBST for the biointerface with the specific guiding sequence GS (red bars) and the random guiding sequence (grey bars).

4. Fluorescence microscopy

The fluorescence images were acquired by confocal fluorescence microscopy after the RCA reaction and labeling with Cy5-LS. They were processed by the ImageJ software in order to determine the number of attached ssDNA chains, manifesting themselves as individual bright spots. By using a threshold, binary image was defined with identified high intensity area (Figure S3) that were subsequently counted (spots exhibited area smaller than 10 μm^2 Figure S3b). The average distance between the spots were determined as square root of the whole image area divided by the number of counted spots.

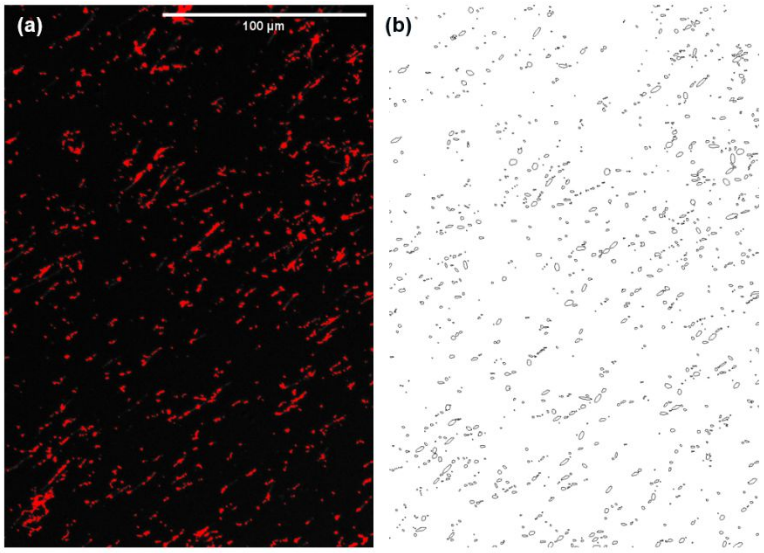

Figure S3. a) Analysis by the ImageJ software for a) determining the color threshold and b) counting the number of spots, shown for a plasmonic sensor chip with RCA-generated ssDNA from c = 400 fM.

5. Gel-electrophoresis

The 0.8% agarose gel electrophoresis was performed to prove the presence of RCA product in solution (not on the surface) after the reaction time of *t* = 30 s, 1 min, 2 min, 5 min, 10 min, 20 min. The RCA reaction mix contained 20 µL of circularized padlock probe with a molar concentration of 40 nM, 2 µL of biotin/20T/TS- with the molar concentration of 40 nM as primer, 1 µL of dNTPs with molar concentration of 25 µM and 20 Units of ϕ29-Polymerase with 13 µL NFW-BSA (0.2 mg/mL). The samples were incubated on the HulaMixer for the indicated time at room temperature and inactivated on the thermomixer at 70°C and 700 rpm for 10 minutes. Afterwards, 10 µL of the reaction mixture with 10 µL of 1:10 diluted loading dye were loaded in the wells of the agarose-gel and separated according to size at 100 V for about 30 to 40 minutes.

As shown in Figure S4a. series of dark bands becomes visible, located at higher kb-values with increasing reaction time *t*, which can be associated with the RCA-generated ssDNA. From the width of the bands a distribution of the RCA-generated DNA length can be estimated, also reported in previous publications. For longer RCA-times, the length of ssDNA exceeds the range that can be quantified by using the used ladder (shown on the left side). The speed of prolongation when the RCA is carried out in the bulk solution can be estimated above 10^3 nt/min, which is higher than 215 nt/min determined on the surface in our previous work.[12] Figure S4b shows the RCA reaction in solution after 10 min in comparison with the padlock probe (*c* = 40 nM). As the circular DNA probe has only 81 nt, the dark band is located at lower bp values when compared to the 100 bp ladder. The intensity of the dark band is higher for the RCA product which can be associated to a high amount of long ssDNA strands.

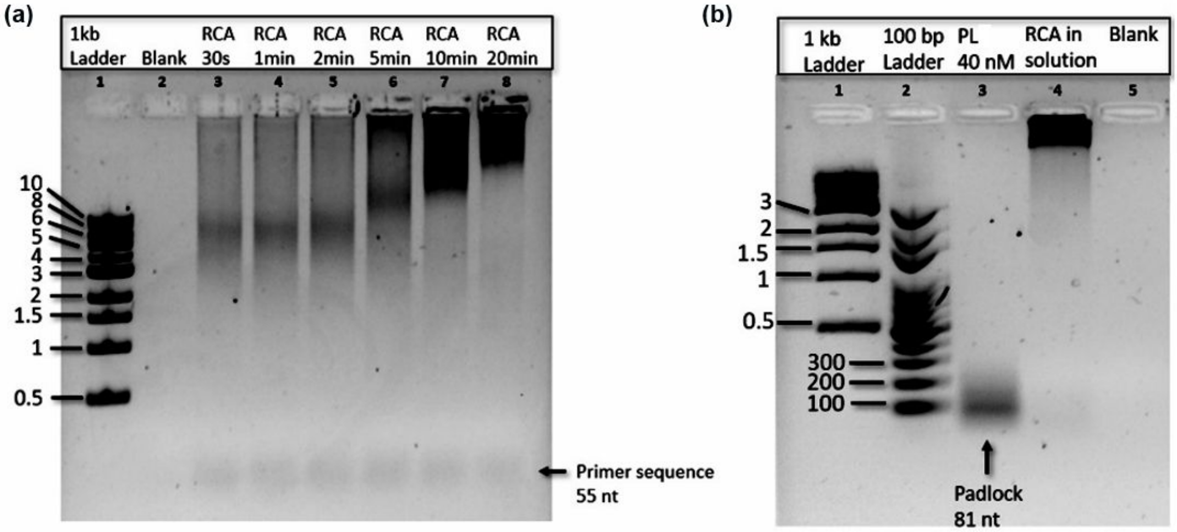

Figure S4. Agarose gel-electrophoresis: a) lane 1) 1kb ladder; lane 2) randomized sequence instead of target sequence (TS-) with RCA reaction of 20 mins Lane 3 to 8) molar concentration of padlock probe 40 nM with RCA reaction times of 30 s, 1 min, 2 min, 5 min, 10 min, 20 min. b) lane 1) 1kb ladder; lane 2) 100 bp ladder; lane 3) padlock probe with molar concentration *c* = 40 nM; lane 4) RCA product after 10 min.

6. Atomic force microscopy

The RCA protocol was carried out on the SPR sensor chips surface in the flow cell for the same conditions as used for the optical measurement. The assay was carried out for the concentration of the padlock probe of 400 fM. Figure S5 shows the topography acquired by the AFM (see methods part in the manuscript) for the surface without and with the specific guiding sequences immobilized.

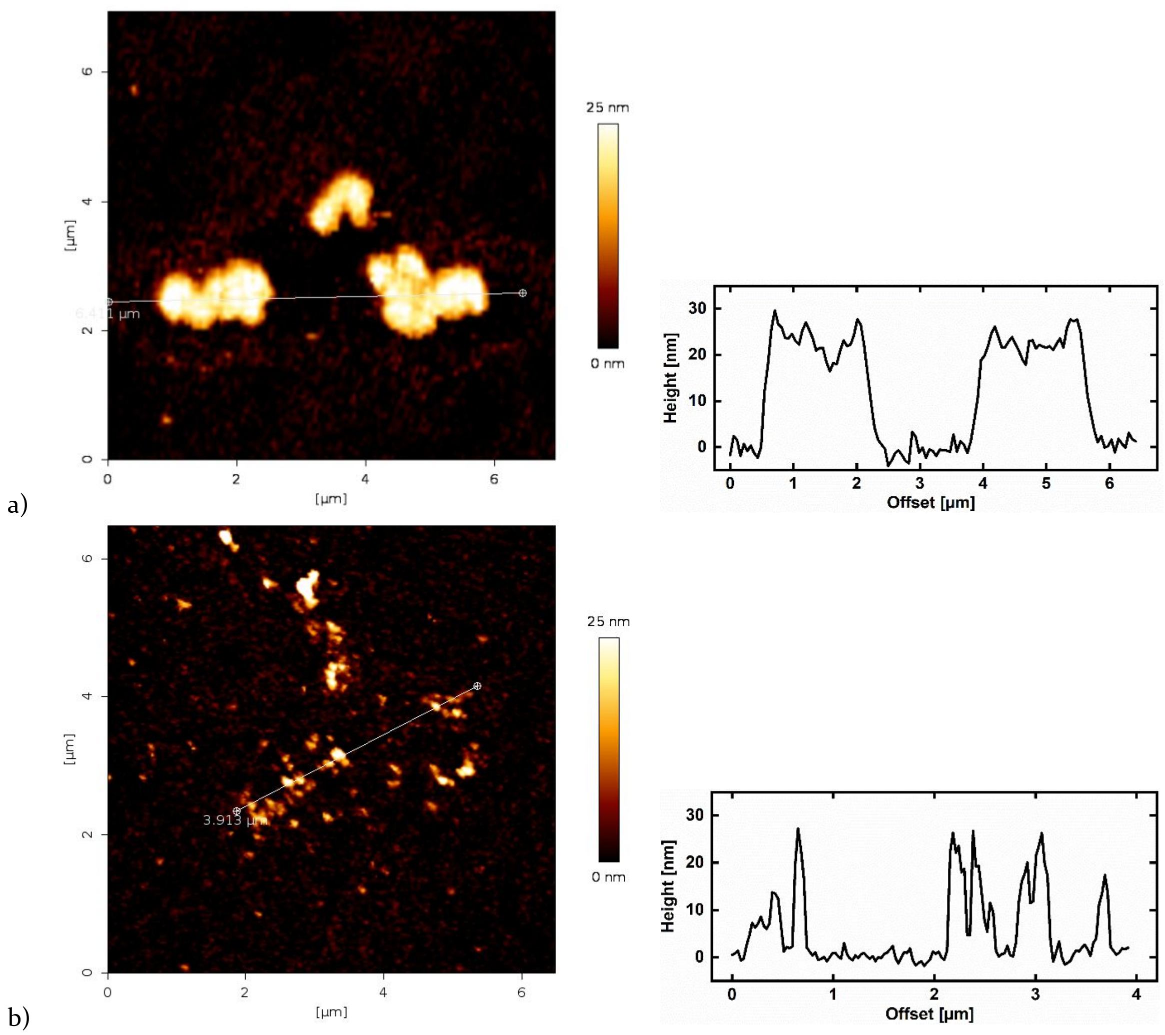

Figure S5. Topography of RCA chains on the surface of SPR sensor chip observed for biointerface with a) the control scrambled ssDNA guiding sequences b) without the ssDNA guiding sequences.

REFERENCES

1. Kühnemund, M., Witters, D., Nilsson, M. & Lammertyn, J. Circle-to-circle amplification on a digital microfluidic chip for amplified single molecule detection. *Lab Chip* **14**, 2983–2992 (2014).

2. Wang, C. *et al.* DNA microarray fabricated on poly(acrylic acid) brushes-coated porous silicon by in situ rolling circle amplification. *Analyst* **137**, 4539 (2012).

3. Sato, K. *et al.* Microbead-based rolling circle amplification in a microchip for sensitive DNA detection. *Lab Chip* **10**, 1262 (2010).

4. Chen, H. *et al.* Detection of vascular endothelial growth factor based on rolling circle amplification as a means of signal enhancement in surface plasmon resonance. *Biosens Bioelectron* **61**, 83–87 (2014).

5. Xiang, Y. *et al.* Isothermal detection of multiple point mutations by a surface plasmon resonance biosensor with Au nanoparticles enhanced surface-anchored rolling circle amplification. *Biosens Bioelectron* **49**, 442–449 (2013).

6. He, P., Liu, L., Qiao, W. & Zhang, S. Ultrasensitive detection of thrombin using surface plasmon resonance and quartz crystal microbalance sensors by aptamer-based rolling circle amplification and nanoparticle signal enhancement. *Chem. Commun.* **50**, 1481–1484 (2014).

7. Yao, C., Xiang, Y., Deng, K., Xia, H. & Fu, W. Sensitive and specific HBV genomic DNA detection using RCA-based QCM biosensor. *Sens Actuators B Chem* **181**, 382–387 (2013).

8. de la Torre, T. Z. G., Herthnek, D. & Strømme, M. A Magnetic Nanobead-Based Read-Out Procedure for Rapid Detection of DNA Molecules. *J Nanosci Nanotechnol* **17**, 2861–2864 (2017).

9. Yi, X., Li, L., Peng, Y. & Guo, L. A universal electrochemical sensing system for small biomolecules using target-mediated sticky ends-based ligation-rolling circle amplification. *Biosens Bioelectron* **57**, 103–109 (2014).

10. Tang, X. *et al.* Strand displacement-triggered G-quadruplex/rolling circle amplification strategy for the ultra-sensitive electrochemical sensing of exosomal microRNAs. *Microchimica Acta* **187**, 172 (2020).

11. Wang, Q. *et al.* Coupling of background reduction with rolling circle amplification for highly sensitive protein detection via terminal protection of small molecule-linked DNA. *Analyst* **138**, 5751 (2013).

12. Lechner, B. *et al.* In Situ Monitoring of Rolling Circle Amplification on a Solid Support by Surface Plasmon Resonance and Optical Waveguide Spectroscopy. *ACS Appl Mater Interfaces* **13**, (2021).
